# Two Cases of Diffuse Duodenitis Associated with Ulcerative Colitis

**DOI:** 10.1155/2012/396521

**Published:** 2012-10-17

**Authors:** Katsuya Endo, Masatake Kuroha, Hisashi Shiga, Yoichi Kakuta, Seiichi Takahashi, Yoshitaka Kinouchi, Tooru Shimosegawa

**Affiliations:** Division of Gastroenterology, Tohoku University Graduate School of Medicine, 1-1 Seiryo, Aoba-ku, Sendai 980-8574, Japan

## Abstract

The upper gastrointestinal tract is not generally considered a target organ in ulcerative colitis (UC). However, several cases showing upper gastrointestinal involvement in UC have been reported. In this report, we present 2 rare cases of diffuse duodenitis accompanying pancolonic UC. Case patient 1 was a 44-year-old man who developed diffuse duodenitis shortly after colectomy and was successfully treated with mesalazine. Case patient 2 was a 25-year-old woman who developed diffuse duodenitis under a steroid-free condition and was successfully treated with prednisolone. The 2 patients had *Helicobacter pylori*-negative duodenitis that resembled colonic lesions of UC in both the endoscopic and histological findings. No evidence of Crohn's disease was found in these cases. We diagnosed both cases as typical UC-associated diffuse duodenitis. The occurrence of gastrointestinal involvement in UC has been attracting attention because such lesions could potentially open a new window for studying the etiology and pathogenesis of UC. Further studies involving a large number of patients are needed to clarify whether the upper gastrointestinal tract is a target organ in UC.

## 1. Introduction

Ulcerative colitis (UC) is a chronic inflammatory disease of the colon that has an unknown etiology. Although UC is known to have various extracolonic manifestations, the upper gastrointestinal tract is not generally considered a target organ. However, several case reports describing upper gastrointestinal involvement in UC have been published [1–4]. These reports described gastritis or diffuse duodenitis that resembled colonic lesions of UC in both the endoscopic and pathological findings. Recently, some case series and case-control studies reported that gastroduodenal involvement in UC may be more frequent than previously estimated [5–8]. The occurrence of gastrointestinal involvement in UC has been attracting attention because such lesions could potentially open a new window for studying the etiology and pathogenesis of UC.

In this report, we describe our experience of 2 rare cases of diffuse duodenitis associated with UC.

## 2. Case Presentation 

### 2.1. Case 1

**Figure 1:**
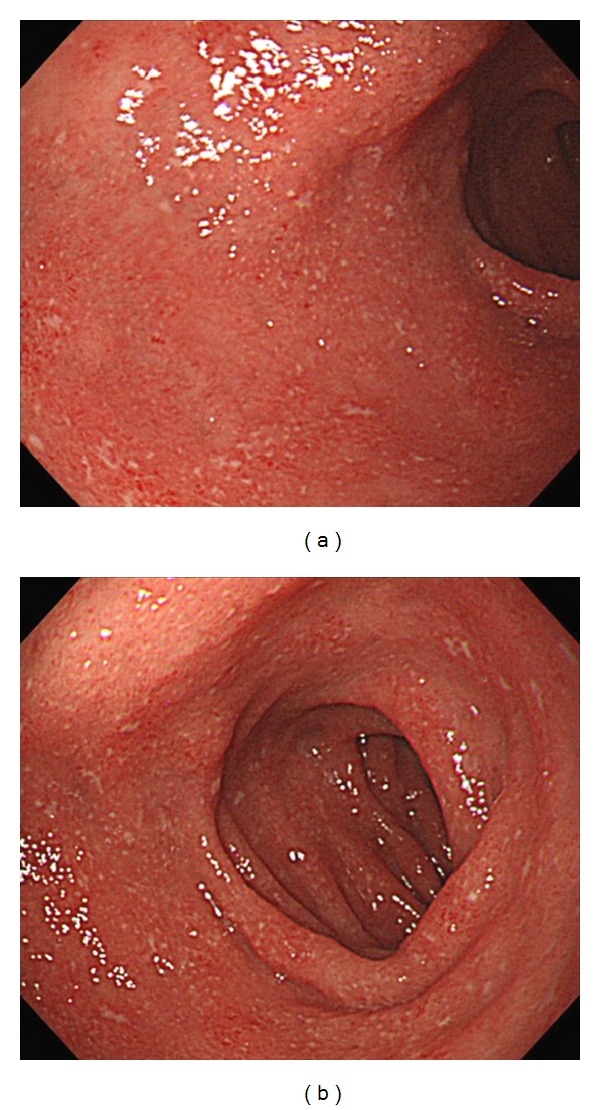
(a) Endoscopy of the upper gastrointestinal tract showed multiple small erosions and diffuse granular changes in the duodenal bulb that were similar in appearance to colonic lesions of UC. (b) Endoscopic findings of the descending portion of the duodenum also showed multiple small erosions and diffuse granular changes.

**Figure 2:**
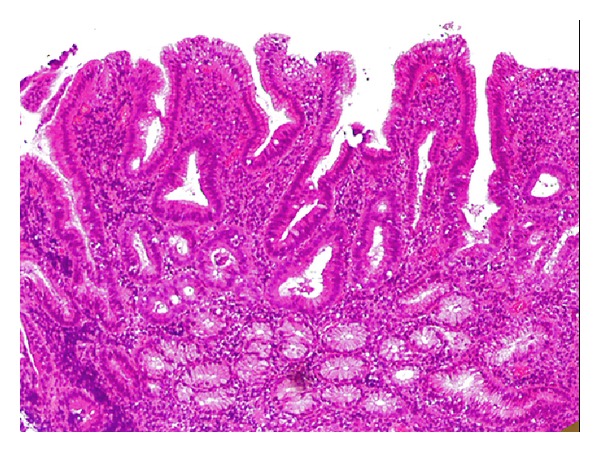
Histological analysis of the duodenal lesions revealed a decrease of goblet cells and severe chronic inflammation without granulomas.

A 44-year-old man with a 4-year history of pancolonic UC underwent laparoscopy-assisted proctocolectomy because his disease was severe and steroid resistant. No evidence of Crohn's disease was found at the time of surgery, and the gross and microscopic features of the colectomy specimen were those of typical pancolonic UC. On the 10th day after the operation, he complained of epigastralgia and tarry stool. Endoscopy of the upper gastrointestinal tract showed multiple small erosions and diffuse granular changes in the bulb and the descending portion of the duodenum that were similar in appearance to colonic lesions of UC (Figure 1). There were no abnormal findings in the stomach, including the gastric antrum. Histological examinations of biopsies from the duodenal lesions revealed a decrease of goblet cells and severe chronic inflammation with lymphoplasmacytic infiltration (Figure 2). Neither granuloma nor *Helicobacter pylori* (*H. pylori*) was detected in the biopsy specimen. Mesalazine administration dramatically improved the symptoms, although the endoscopic findings did not show substantial change. He continues to be observed at our hospital as an outpatient while continuing mesalazine treatment. He has not developed any Crohn's-like complications such as fistulas, sinus tracts, or strictures during the follow-up time of about 4 years. 

### 2.2. Case 2

**Figure 3:**
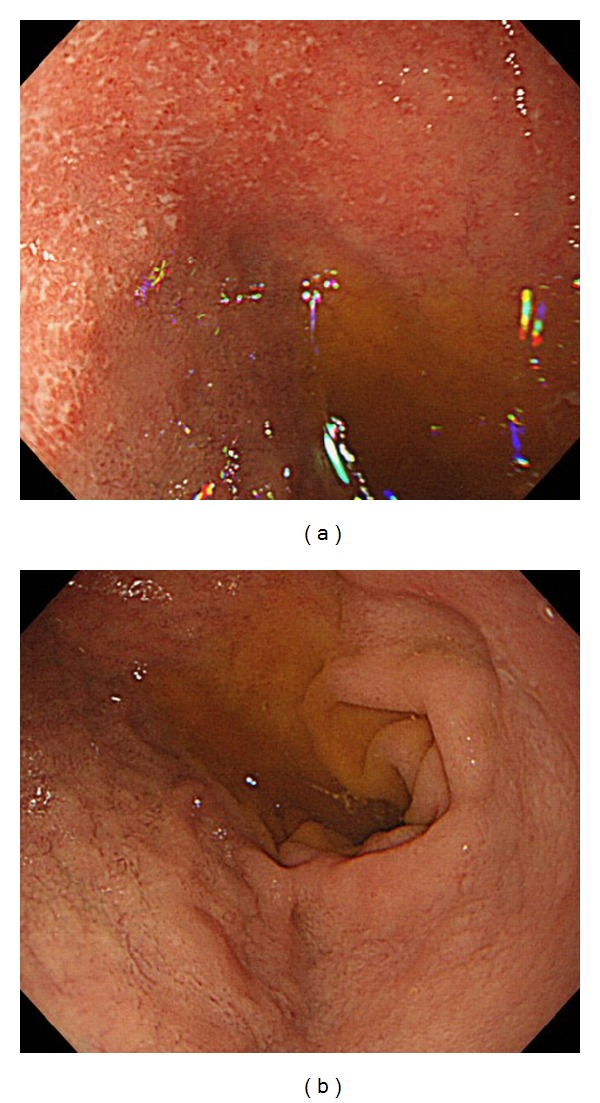
(a) Endoscopy of the upper gastrointestinal tract showed multiple small erosions in the duodenal bulb that were similar in appearance to colonic lesions of UC. (b) Endoscopic findings of the descending portion of the duodenum showed diffuse granular changes.

**Figure 4:**
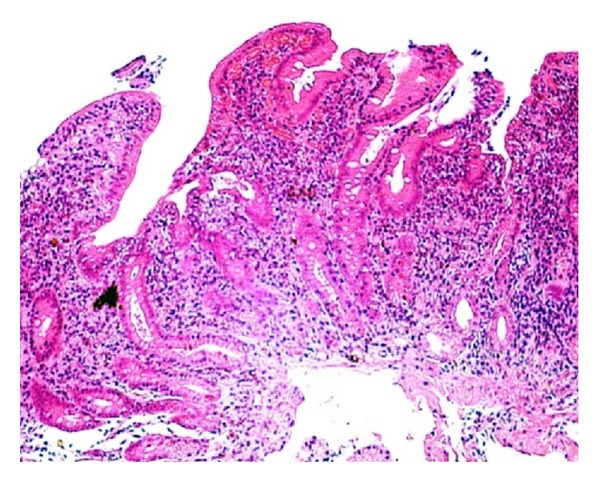
Histological analysis of the duodenal lesions revealed a decrease of goblet cells and severe chronic inflammation cryptitis without granulomas.

A 25-year-old woman with a 1-year history of pancolonic UC was started on oral and topical mesalazine treatment as an outpatient in our hospital because her disease was in the moderately active phase. There was no clinical, radiographic, or endoscopic evidence of Crohn's disease at this time. Before starting the treatment, she also complained of upper abdominal discomfort. Therefore, we performed endoscopy of the upper gastrointestinal tract, which showed multiple small erosions in the duodenal bulb and diffuse granular changes in the descending portion of the duodenum that were similar in appearance to colonic lesions of UC (Figure 3). There were no abnormal findings in the stomach including the gastric antrum. Histological examinations of biopsies from the duodenal lesions revealed a decrease of goblet cells, severe chronic inflammation with lymphoplasmacytic infiltration, and cryptitis (Figure 4). Neither granuloma nor *H. pylori* was detected in the biopsy specimen. Although the symptoms of UC (bloody diarrhea and lower abdominal pain) improved after administration of oral and topical mesalazine, her upper abdominal discomfort did not improve despite administration of a proton pump inhibitor (PPI). However, additional administration of oral prednisolone promptly improved the upper abdominal discomfort. Oral prednisolone was gradually tapered off and completely stopped after 2 months. She is now under continuous observation at our hospital while continuing mesalazine treatment. She has not developed any Crohn's-like complications such as fistulas, sinus tracts, or strictures during the follow-up time of about 4 years. 

## 3. Discussion

Although upper gastrointestinal lesions in patients with UC are generally considered rare, some case reports describing upper gastrointestinal involvement in UC have been published. Especially for duodenal lesions, several case reports have described the characteristics of diffuse duodenitis associated with UC [1–3, 9]. 

UC-associated diffuse duodenitis is characterized by duodenal lesions that closely resemble UC lesions in the colon in both the endoscopic and pathological findings [1–3, 5]. UC-associated diffuse duodenitis is often seen in the bulb and/or the descending and/or the horizontal portion of the duodenum, and endoscopic findings show multiple erosions, aphthae (multiple white spots surrounded by a red halo), ulcers, and granular changes that are similar in appearance to colonic lesions of UC. Pathological findings also resemble the features of colonic lesions of UC, including chronic inflammation without any granulomas, a decrease of goblet cells, cryptitis, and crypt abscess. Moreover, UC-associated diffuse duodenitis is probably independent from *H. pylori* infection because many of the previously reported cases were *H. pylori* negative [1, 2]. In the present report, our 2 patients also had *H. pylori*-negative duodenal lesions resembling colonic lesions of UC in both the endoscopic and pathological findings. Moreover, no clinical or pathological evidence of Crohn's disease was seen in both cases. Therefore, our present cases were compatible with the distinctive characteristics described above, and diagnosed as typical UC-associated diffuse duodenitis.

Little is known about the trigger of the onset of UC-associated diffuse duodenitis. Hori et al. reported that a lower dose of prednisolone was a significant risk factor for developing UC-associated gastroduodenitis [6]. In fact, duodenitis occurred under a steroid-free or steroid-withdrawal status in many previous cases [2, 3]. The duodenitis in our cases also developed under steroid-free conditions. Hori et al. also reported that the presence of pancolitis was also a significant risk factor for UC-associated gastroduodenitis [6]. In the report of Hisabe et al., all of the 15 patients with UC-associated gastroduodenal lesions had accompanying pancolitis or developed duodenitis after colectomy [5]. In fact, many of the reported cases of UC-associated diffuse duodenitis were accompanied by pancolonic UC or developed after colectomy [1–3, 5]. In the present report, the 2 patients had pancolonic UC and case patient 1 developed duodenitis after colectomy. Here, attention should be paid to the interval between colectomy and the onset of duodenitis. Case patient 1 developed diffuse duodenitis shortly after proctocolectomy. Some similar cases of UC-associated duodenitis or enteritis that developed shortly after colectomy have been reported. Rubenstein et al. reported about a 38-year-old man who developed diffuse, severe enteritis shortly after undergoing colectomy for UC, and reviewed some similar cases [10]. Nakajima et al. also reported a case of diffuse duodenitis and enteritis following total colectomy for UC, which was very similar to our case 1 [11]. Our cases and these previous reports suggest that UC-associated duodenitis and enteritis might be frequent occurrences in the period shortly after colectomy.

No consensus exists about the treatment for gastroduodenitis accompanying UC. The reported cases of gastroduodenitis showed no response to PPIs. However, several reports described that mesalazine or sulfasalazine were very effective [2, 4]. In our case 1, administration of mesalazine dramatically improved the symptoms. Corticosteroids were also reported to be effective for gastroduodenitis accompanying UC [3, 4, 9, 12]. In our case 2, although neither mesalazine nor PPI administration was effective, additional administration of oral prednisolone promptly improved the upper abdominal discomfort in the patient. These previous reports and our experiences indicate that upper gastrointestinal lesions occurring with UC may be treated with the traditional therapies for UC such as mesalazine or corticosteroids. We propose sulfasalazine or mesalazine as the first-choice treatment and corticosteroids as the second choice. 

Recently, some case series and case-control studies described that gastroduodenal involvement in UC may be more frequent than previously estimated [5–8]. Because the inflammation of UC may not be restricted to the large intestine, it is important to examine the upper gastrointestinal tract to confirm whether UC-associated gastritis or duodenitis exists. 

Further studies involving a large number of patients are needed to clarify whether the upper gastrointestinal tract is a target organ in UC and to better understand the etiology and pathogenesis of UC.
